# High-density lipoprotein cholesterol and multiple myeloma: A systematic review and meta-analysis

**DOI:** 10.1016/j.athplu.2023.09.003

**Published:** 2023-09-21

**Authors:** Anastasios Makris, Antonia Pagkali, Emmanouil Nikolousis, Theodosios D. Filippatos, Aris P. Agouridis

**Affiliations:** a School of Medicine, European University Cyprus, Nicosia, Cyprus; b School of Medicine, National and Kapodistrian University of Athens, Greece; c Department of Internal Medicine, School of Medicine, University of Crete, Heraklion, Greece; d Department of Internal Medicine, German Oncology Center, Limassol, Cyprus

**Keywords:** Multiple myeloma, High-density lipoprotein cholesterol, HDL-C, Lipid profile

## Abstract

**Background and aims:**

To systematically investigate all relevant evidence on the association between high-density lipoprotein cholesterol (HDL-C) and multiple myeloma (MM).

**Methods:**

We searched PubMed and Cochrane library databases (up to 20 September 2022) for studies with evidence on HDL-C in patients with MM. A qualitative synthesis of published prospective and retrospective studies for the role of HDL-C and other lipid profile parameters in MM was performed. Additionally, a meta-analysis on HDL-C mean differences (MD) between MM cases and controls was performed.

**Results:**

Fourteen studies (3 prospective, 11 retrospective) including 895 MM patients were eligible for this systematic review. Ten studies compared HDL-C levels in MM patients with healthy controls. In these 10 studies (n = 17,213), pooled analyses showed that MM patients had significantly lower HDL-C levels compared to healthy controls (MD: −13.07 mg/dl, 95% CI: −17.83, −8.32, p < 0.00001). Regarding secondary endpoints, total cholesterol (TC) (MD: −22.19 mg/dl, 95% CI: −39.08, −5.30) and apolipoprotein A-I (apoA-I) (−40.20 mg/dl, 95% CI: −55.00, −25.39) demonstrated significant decreases, while differences in low-density lipoprotein cholesterol (LDL-C) (MD: −11.33 mg/dl, 95% CI: −36.95, 14.30) and triglycerides (MD: 9.93 mg/dl, 95% CI: −3.40, 23.26) were not shown to be significant.

**Conclusions:**

HDL-C, as well as TC and apoA-I, levels are significantly decreased in MM. Hence, lipid profile parameters should be taken into account when assessing such patients.

## Highlights

• Low high-density lipoprotein cholesterol (HDL-C) levels represent an important feature in multiple myeloma (MM).
• Patients with MM have lower HDL-C, total cholesterol, and apolipoprotein A-I levels compared with healthy controls.
• Lipid profile parameters should be taken into account when assessing MM patients.

## 1 Introduction

Multiple myeloma (MM) or plasma cell myeloma is the second most common hematological malignancy, characterized by uncontrolled monoclonal proliferation of plasma cells in the bone marrow [1,2]. According to the most recent criteria by the International Myeloma Working Group (IMWG), predominant biomarkers of MM include plasma cell infiltration of the bone marrow, CRAB features (i.e., hypercalcemia, renal failure, anemia, bone lesions on imaging), serum free light chain ratio, MRI lesions, and monoclonal (M) protein [3].

Arguably, MM is a heterogeneous entity with variations in many clinical and laboratory parameters, including those in lipid profile and, especially, in high-density lipoprotein cholesterol (HDL-C). High-density lipoproteins (HDL) have many protective properties including anti-oxidative, anti-apoptotic, and anti-inflammatory effects [4,5]. HDL and their major constituent, apolipoprotein A-I (apoA-I), inhibit hematopoietic stem and progenitor cell proliferation [6]. Consequently, low HDL-C and/or apoA-I levels are associated with an increased risk of hematological cancers like MM [7,8], while they may also promote cancer progression and resistance to therapy [9]. However, both null and positive associations between HDL-C and cancer have been described [10]. Other lipoproteins, including low density lipoprotein cholesterol (LDL-C), total cholesterol (TC) and triglycerides (TGs), have also been linked to MM [11,12].

To date, the association between hypoalphalipoproteinemia and MM has not been thoroughly studied, despite relevant supportive evidence. Herein, we performed a systematic review and meta-analysis to investigate the value of HDL-C and other lipid parameters as biomarkers in MM disease.

## 2 Materials and methods

This systematic review has been registered in PROSPERO (ID number: CRD42022363568) and adheres to the Preferred Reporting Items for Systematic Reviews and Meta-Analyses (PRISMA) 2020 statement (PRISMA Statement, Ottawa, ON, Canada) [13].

In the present study, the primary outcome was to systematically investigate the association between MM disease and HDL-C levels. Therefore, all studies with available evidence on HDL-C and MM were eligible for inclusion. Our secondary outcome was to assess the association of MM with other parameters of lipid metabolism, including LDL-C, TC, TGs and apoA-I.

### 2.1 Search strategy

We searched PubMed and Cochrane library databases up to 20^th^ of September 2022 using the following keywords: (High-density Lipoprotein Cholesterol OR HDL) AND (Multiple Myeloma).

### 2.2 Study design

We performed qualitative and quantitative syntheses of both prospective and retrospective studies to evaluate the role of HDL-C levels and, secondarily, of other lipid profile parameters (i.e., LDL-C, TC, TGs and apoA-I) in MM disease.

### 2.3 Screening and eligibility

Deduplication was performed by use of Zotero reference managing software. Screening for eligible articles involved two steps: initially, one author (AM) excluded non-eligible studies by screening titles and abstracts and evaluated the remaining full-text articles for eligibility. Then, another author (APA) confirmed these decisions. Disagreements were resolved by consensus. Eligibility criteria followed the PICOS (population, intervention, comparators/controls, outcomes, and study design) study question format, as follows:

Population: Patients with MM disease.

Intervention: Measurement of lipid levels.

Comparator/controls: Healthy participants (where applicable).

Outcomes: Mean differences of HDL-C and other lipoprotein levels between MM patients and controls.

Study design: Both observational studies and clinical trials were included.

Studies not meeting the eligibility criteria were excluded.

### 2.4 Data extraction

Studies were assessed independently by two authors (AM, APA) and study characteristics were extracted. Discrepancies between reviewers were resolved by consensus. Data extraction adhered to the PRISMA guidelines. Authors scrutinized both references of eligible and related articles to identify undetected studies.

### 2.5 Methodological assessment of included studies

The Newcastle-Ottawa Scale (NOS) was applied individually for the assessment of all included studies [14]. NOS assesses 3 main criteria categories, which are the following: selection, comparability, and exposure. Selection and comparability are further divided into subcategories, which allow a more in-depth assessment. The maximum score that can be given to each study is 9. A score of 7–9 indicates high quality studies, a score of 4–6 indicates high risk for bias, and a score of 0–3 shows very high-risk for bias. Risk of bias for each study was evaluated independently by AM and AP, and discrepancies were resolved by consensus.

### 2.6 Data analysis

Where sufficient information was obtainable and the outcome measures were comparable, meta-analyses were performed, allowing a quantitative analysis of studies. The pooled estimations regarding outcomes were expressed as continuous. Our meta-analysis was performed using a random effects model or a fixed effects model. For continuous data, mean differences (MD) and 95% confidence intervals (CΙs) were calculated. Statistical analysis was performed using the Review Manager (RevMan) Version 5.0 software (The Nordice Cochrane Center, The Cochrane Collaboration, Copenhagen, Denmark, 2008). P < 0.05 was considered significant.

### 2.7 Heterogeneity analysis

The existence of statistical heterogeneity among included studies was assessed using the I^2^ test. The heterogeneity was considered low, moderate or high if the I^2^ was 25%, 50% or >75% respectively. If the p-value was less than 0.10, the random effects model was adopted, and vice versa. The inter-trial heterogeneity was assessed using the Q test and the I^2^ statistic.

## 3 Results

### 3.1 Study selection

**Fig. 1:**
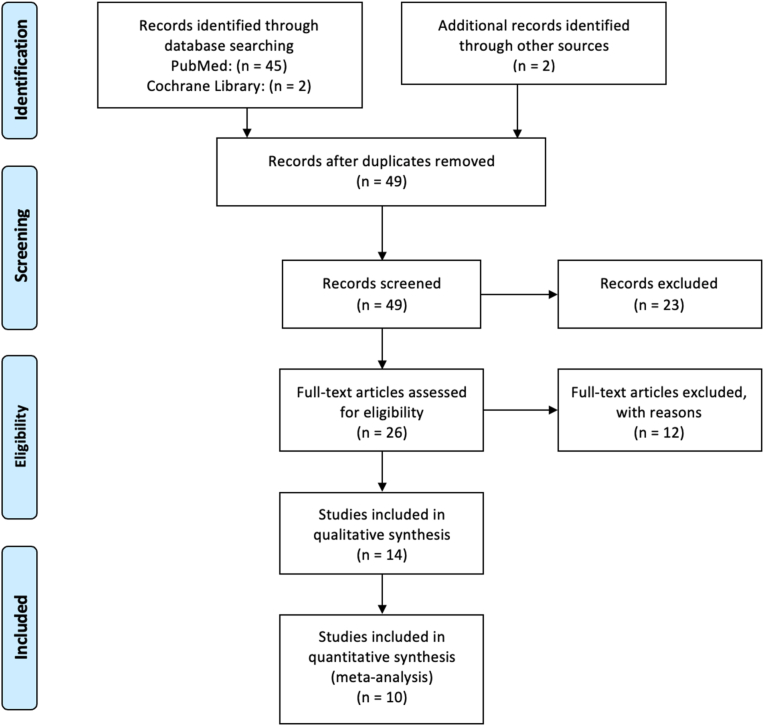
Literature search and study selection by use of PRISMA flowchart.

The study selection process is depicted in the PRISMA flowchart (Fig. 1). Upon database search, we identified 49 records and finally, 14 studies met the inclusion criteria [[15], [16], [17], [18], [19], [20], [21], [22], [23], [24], [25], [26], [27], [28]]. Excluded studies [7,11,[29], [30], [31], [32], [33], [34], [35], [36], [37], [38]] together with the reasons of exclusion, are shown in Supplementary Table S1.

### 3.2 Study characteristics

All 14 studies were performed between 1987 and 2021. Out of them, 11 studies were retrospective, while 3 were prospective. Specifically, 3 studies were conducted in Turkey [16,17,28] 2 were conducted in France [19,20], 2 in Iran [15,18], 2 in China [26,27], 2 in Poland [23,24], and the rest took place in Brazil, Israel, and the U.S [21,22,25]. In total, they included 17,677 participants, 895 of which were MM patients. The significantly larger number of controls compared to MM cases is mostly attributed to the population study by Kabat et al. where the non-case group consisted of 16,366 women [22]. Details of the included studies are available in Table 1. Mean ages, where available, were similar between groups. All included case-control studies were matched by sex. In the 14 studies included in the present review, HDL-C levels of patients with active disease ranged from 26 to 52.4 mg/dl. On the other hand, HDL-C levels of controls were found to be in the range of 38.6–58 mg/dl. Finally, two of the included studies, both conducted by Ellidag and colleagues, use the same set of MM patients. Of note, estimates provided in one of these studies [17] represented median values.

**Table 1:** Characteristics of eligible studies.

First author	Year	Country	Study Design	MM Patients (n)	MM HDL-C (mg/dL)
Dehghani [15]	2020	Iran	Cohort	23	41 ± 6.9
Ellidag [16]	2014	Turkey	Case-Control	40	35 ± 13
Ellidag [17]	2014	Turkey	Cohort	40	30 (20–41)^a^ (FLCe-MM)^a^
					38 (33–42)^a^ (NFLCe-MM)^a^
Faridvand [18]	2016	Iran	Case-Control	34	38.00 ± 3.52
Hachem [19]	1987	France	Case-Control	43	41.38 ± 19.33
Hachem [20]	1988	France	Case-Control	7	26
Hungria [21]	1999	Brazil	Case-Control	20	49.2 ± 12.7
Kabat [22]	2018	United States	Clinical Trial, Cohort	112	52.4 ± 11.5
Kuliszkiewicz-Janus [23]	1995	Poland	Case-Control	20	26.68 ± 6.574
Kuliszkiewicz-Janus [24]	2008	Poland	Case-Control	32	50 ± 13 (at diagnosis)
					37 ± 12 (during active disease)
					55 ± 18 (during remission)
Levy [25]	1984	Israel	Case Control	21	40.9 ± 12
Liang [26]	2019	China	Cohort	307	35.96 ± 13.148
Lin [27]	2021	China	Cohort	94	46.4 (23.2–96.67) (NLCCN-MM)^a^
					30.936 (15.468–58) (LCCN-MM)^a^
Yavasoglu [28]	2008	Turkey	Case-Control	102	42 ± 17

FLCe-MM: free light chain excretion multiple myeloma; High-Density Lipoprotein Cholesterol; LCCN-MM: light chain cast nephropathy multiple myeloma; NFLCe-MM: non-free light chain excretion multiple myeloma; NLCCN-MM: non-light chain cast nephropathy multiple myeloma; N/A: Not Available.

a Median values.

### 3.3 Study outcomes – meta-analysis

**Fig. 2:**
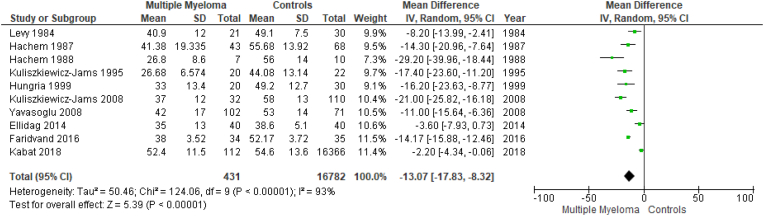
Forest plot of comparison: Multiple Myeloma vs Controls, outcome: High-density lipoprotein cholesterol (mg/dl).

**Fig. 3:**
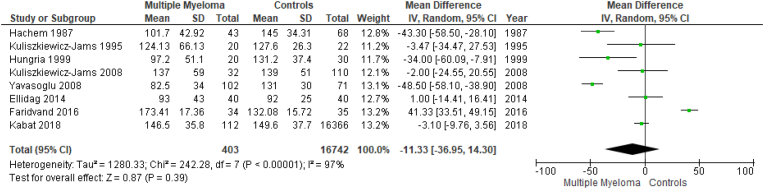
Forest plot of comparison: Multiple Myeloma vs Controls, outcome: Low-density lipoprotein cholesterol (mg/dl).

**Fig. 4:**
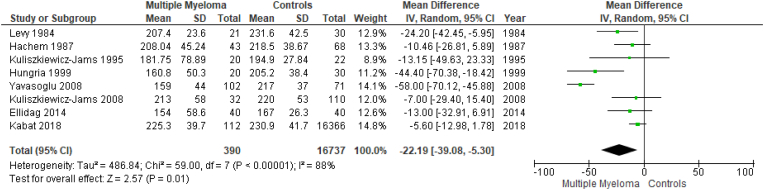
Forest plot of comparison: Multiple Myeloma vs Controls, outcome: Total cholesterol (mg/dl).

**Fig. 5:**
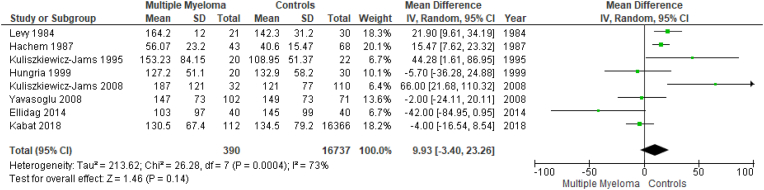
Forest plot of comparison: Multiple Myeloma vs Controls, outcome: Triglycerides (mg/dl).

**Fig. 6:**
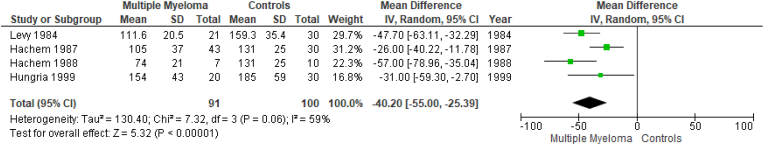
Forest plot of comparison: Multiple Myeloma vs Controls, outcome: Apolipoprotein A-I (mg/dl).

**Fig. 7:**
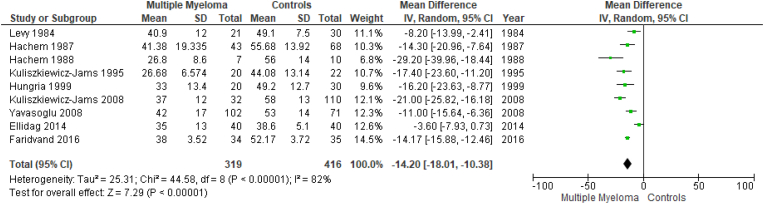
Forrest plot of comparison: Multiple Myeloma vs Controls, excluding Kabat 2018 study, outcome: High-density lipoprotein cholesterol (mg/dl).

Results are summarized in Fig. 2, Fig. 3, Fig. 4, Fig. 5, Fig. 6, Fig. 7. To perform our meta-analysis regarding HDL-C, LDL-C, TC, TGs and apoA-I we used 10, 8, 8, 8 and 4 studies, respectively. Our quantitative synthesis revealed significant decreases in the primary endpoint, HDL-C (MD: −13.07 mg/dl, 95% CI: −17.83, −8.32, I^2^: 93%, p < 0.00001), (10 studies, n = 17,213). Regarding secondary endpoints, TC (MD: −22.19 mg/dl, 95% CI: −39.08, −5.30, I^2^:88%, p = 0.01), (8 studies, n = 17,127) and apoA-I (MD: −40.20 mg/dl, 95% CI: −55.00, −25.39, I^2^:59%, p < 0.00001), (4 studies, n = 191) demonstrated significant decreases, while changes in LDL-C (MD: −11.33 mg/dl, 95% CI: −36.95, 14.30, I^2^:97%, p = 0.39), (8 studies, n = 17,145) and TGs (MD: 9.93 mg/dl, 95% CI: −3.40, 23.26, I^2^:73%, p = 0.14), (8 studies, n = 17,127) were not significant. Due to the use of only females in the study by Kabat et al., a second meta-analysis on HDL-C was performed excluding this study (Fig. 7). The association between HDL-C and MM remained significant (MD: −14.20 mg/dl, 95% CI: −18.01, −10.38, I^2^: 82%, p < 0.00001), (9 studies, n = 735).

### 3.4 Quality appraisal and publication bias

The NOS for quality assessment of observational studies yielded the following results in regard to the included studies. The mean value for the 14 observational (case control, cohort) studies included in this review was 7.21 points (2 studies with 9 points; 3 studies with 8 points; 6 studies with 7 points; 2 studies with 6 points, and 1 study with 5 points). As per the aforementioned scoring system, this result indicates that, overall, the included studies are of high quality (Supplementary Table S2 and Table S3).

## 4 Discussion

This systematic review focuses on the association between HDL-C levels and MM disease. Our results suggest that patients with MM have significantly lower HDL-C levels when compared with controls. Regarding the rest of the lipid profile parameters, the greatest reductions were observed in apoA-I, followed by those in TC. Similar findings were previously obtained by studies assessing cancer patients in general, although inter-disease differences were noticed [39].

Large population-based studies have shown that low cholesterol levels are associated with a higher odd of MM presence, while these aberrations may normalize upon achievement of remission [7,22,24,29,40,41]. The mechanisms, however, by which MM patients present with low lipid levels have not been clearly defined. In general, cholesterol is important for cell membrane structure and function in both normal and malignant cells and, as such, it may be utilized by malignant cells for membrane biosynthesis [42]. Moreover, malignancy-related hypolipidemia may be explained by an impairment in the activity of lipoprotein lipase, or it may even be directly attributed to the action of chemotherapy [42]. Furthermore, cancer is also characterized by inflammation, which can reduce HDL-C levels in various ways; namely by the action of endotoxins and cytokines, the suppression of apoA-I production by the liver, the increases in endothelial cell lipase and secretory phospholipase A2, and the impairment in Lecithin:cholesterol acyltransferase (LCAT) function [43]. Similar mechanisms have been proposed for low HDL-C in the setting of infections such as leishmaniasis, leptospirosis, brucellosis, tuberculosis and, recently, coronavirus disease 2019 (COVID-19) [12,[44], [45], [46]]. In addition, inflammation, may also lead to tumorigenesis [31]. Another postulated HDL-C-lowering mechanism involves the production of antibodies against the protein moiety of the HDL particle, which tend to be more common than *anti*-LDL immunoglobulins, despite the higher immunogenicity of LDL [47]. Lastly, MM-related decreases in HDL-C, may also be attributed to interference of paraproteins in the biochemical assay and, thus, they may be artifactual findings [48]. Irrespective of the pathophysiology behind hypolipidemia, it has not yet been elucidated whether low HDL-C and other cholesterol levels lead to tumor growth or if they are a consequence of neoplasia instead [42]. Importantly, increased lipoprotein level variability also acts as a risk factor for MM [31]. Nevertheless, in rare cases, MM has been linked to hyperlipidemia as well [47,[49], [50], [51], [52], [53], [54]].

Furthermore, among MM patients, HDL-C levels tend to be lower in those with active disease compared to those in remission (where complete remission is defined as a combination of negative urine and serum immunofixation, lack of soft tissue plasmacytomas, and fewer than 5% plasma cells in bone marrow aspirates) [2,55,56]. In their 2008 study, Kuliszkiewicz-Janus et al. demonstrated lipid level variations based on disease status; specifically, the lowest levels of HDL-C, LDL-C, as well as TC, were reached during the active phase of the disease, while the highest levels were noted upon disease remission [24]. In the same study, nonetheless, TGs followed an almost inverse pattern, with the highest levels being achieved during the active phase of the disease [24]. Moreover, it is well-known that HDL-C levels tend to be higher in pre-menopausal females compared to males [57,58]. The study by Kabat et al. comprised completely of women; however, a sub-analysis excluding this study, revealed that the correlation between MM and HDL-C remained significant.

Higher, but not extremely high, HDL-C and/or apoA-I levels have been linked to better survival outcomes in cancer and MM patients, partly due to antineoplastic effects of apoA-I [10,26,59]. Additionally, structure and function of the lipoprotein also play an important role when it comes to cancer, alike cardiovascular disease (CVD) [60]. Lipid-lowering therapies, such as statins and potentially fibrates, can be of use in patients with MM, since they reduce both the risk of MM development and cancer-related mortality [[60], [61], [62]]. This also holds true in patients with myelodysplastic syndromes and acute myeloid leukemia [63].

MM is linked to metabolic syndrome, a component of which is low HDL-C levels [64]. Low HDL-C has long been connected to CVD via the well-known “HDL hypothesis” [65]. When it comes to MM patients, cardiovascular risk is higher compared to the general population [66]. The correlation between MM and CVD or major adverse cardiovascular events is especially true in the elderly, who constitute the main demographic group suffering from the disease [[67], [68], [69]]. Moreover, MM-related dyslipidemia may be refractory to traditional lipid-lowering therapies [64], while statins may present a preventative role in MM, partly due to a cytotoxic effect on human myeloma cells [64]. Notably, anti-myeloma drugs like proteasome inhibitors and immunomodulatory drugs have demonstrated synergy with lipid-modulating agents [70], but they have also been associated with several cardiovascular adverse events. Such side effects may, in turn, be further attenuated by existing cardiovascular risk factors or established CVD, as it is often the case in elderly patients [69]. Hence, MM-related dyslipidemia may warrant employment of more intensive hypolipidemic treatment to reach lipid targets.

Several limitations are present in this systematic review. Firstly, some of the included studies were published decades ago, while many studies did not account for disease status or history of treatment. Additionally, there was a lack of reporting on use of lipid-lowering agents in approximately half of the included studies. Moreover, our meta-analysis suffers from a high heterogeneity, as indicated by the I^2^ tool (93%, 97%, 88% and 73% for HDL-C, LDL-C, TC, and TGs, respectively). This may be attributed to the differences in the design and different inclusion/exclusion criteria of each included study. Other factors that can explain the high heterogeneity include differences in outcome measurement methods (direct/indirect), inter-patient variations regarding disease stage (e.g. before/during/after chemotherapy, disease severity) and receipt of steroid, cytotoxic or concurrent lipid-lowering therapy. Lastly, fewer studies with available data on LDL-C, TC, TGs and, especially, apoA-I were included in comparison to HDL-C-related studies, and this may introduce bias to our results.

## 5 Conclusion

Based on the above, it is clear that HDL-C and, secondarily, TC and apoA-I do not only reflect cardiovascular risk [9,71], but also represent important features in MM. Our findings point towards a need for implementation of larger clinical studies which will offer more robust data on the clinical significance of the association between HDL-C and MM, as well as on the clinical value of adding a lipid panel in the assessment of patients with MM. Finally, future research perspectives in MM include investigating the role of lipids as potential biomarkers of disease burden and treatment response.

## Financial support

This research did not receive any specific grant from funding agencies in the public, commercial, or not-for-profit sectors.

## Author contribution

A.M.: Conceptualization, Methodology, Formal analysis, Investigation, Writing - Original Draft, Visualization; A.P.: Formal analysis, Writing – Review & Editing; E.N.: Writing - Review & Editing; T.D.F.: Writing - Review & Editing; A.P.A. Conceptualization, Methodology, Formal analysis, Investigation, Writing - Review & Editing. All authors have read and agreed to the published version of the manuscript.

## Declaration of competing interest

The authors declare that they have no known competing financial interests or personal relationships that could have appeared to influence the work reported in this paper.
