# Supplementary material for: High-density lipoprotein cholesterol and multiple myeloma: A systematic review and meta-analysis
Source: Atheroscler Plus. 2023 Sep 21;54:7–13. doi: 10.1016/j.athplu.2023.09.003 (PMC10539640; doi:10.1016/j.athplu.2023.09.003)
Supplement: Multimedia component 1 [file mmc1.docx]

**High-Density Lipoprotein Cholesterol and Multiple Myeloma: A Systematic Review and Meta-analysis**

**Supplementary Material**

| **Table S1: Excluded studies with reasons of exclusion** | | |
| --- | --- | --- |
| **Author** | **Year** | **Reasons for exclusion** |
| Jeong et al. [1] | 2021 | Insufficient data |
| Tavori et al. [2] | 2020 | Case report |
| Liu et al. [3] | 2020 | Could not be retrieved |
| Pedersen et al. [4] | 2020 | Did not report on outcome |
| Veskovski et al. [5] | 2021 | Did not report on outcome |
| Choi et al. [6] | 2021 | Insufficient data |
| Chen et al. [7] | 2022 | Article in Chinese |
| Tsai et al. [8] | 2005 | Case report |
| Aviram et al. [9] | 1985 | Case report |
| Gozzetti et al. [10] | 2008 | Non-eligible population |
| Wang et al. [11] | 2022 | Insufficient data |
| Ozturk et al. [12] | 2021 | Non-eligible population |

Note. Table S1 mentions the excluded studies with reasons of exclusion according to the eligibility criteria of the systematic search.

| **Table S2. Quality appraisal of the included studies using the Newcastle-Ottawa Scale (NOS) for Case-Control studies** | | | | | | | | | |
| --- | --- | --- | --- | --- | --- | --- | --- | --- | --- |
| **Study** | **Selection** | | | | **Comparability** | **Exposure** | | | **Total score** |
|  | **Case definition** | **Case representativeness** | **Controls selection** | **Controls definition** |  | **Ascertainment** | **Same method of ascertainment** | **Non-response rate** |  |
| Ellidag et al., 2014 | * | * | - | - | ** | * | * | * | 7 |
| Faridvand et al., 2016 | * | * | - | * | ** | * | * | * | 8 |
| Hachem et al., 1987 | * | * | - | - | * | * | * | * | 6 |
| Hachem et al., 1988 | * | * | * | - | ** | * | - | * | 7 |
| Hungria et al., 1999 | * | * | - | - | ** | * | * | * | 7 |
| Kuliszkiewicz-Janus et al., 1995 | * | * | * | - | * | * | * | * | 7 |
| Kuliszkiewicz-Janus et al., 2008 | * | * | - | - | ** | * | * | * | 7 |
| Levy et al., 1984 | * | * | - | - | * | * | * | - | 5 |
| Yavasoglu et al., 2008 | * | * | * | * | ** | * | * | * | 9 |

Note. The mean value for the 9 case-control studies included in this systematic review was 7 points, indicating that the included studies are of high quality.

| **Table S3. Quality appraisal of the included studies using the Newcastle-Ottawa Scale (NOS) for Cohort studies** | | | | | | | | | |
| --- | --- | --- | --- | --- | --- | --- | --- | --- | --- |
| **Study** | **Selection** | | | | **Comparability** | **Exposure** | | | **Total score** |
|  | **Cohort representativeness** | **Non-exposed cohort selection** | **Ascertainment** | **Outcome not present from start** |  | **Outcome assessment** | **Follow-up (1)** | **Follow up (2)** |  |
| Dehghani et al., 2020 | * | * | * | - | * | - | * | * | 6 |
| Ellidag et al., 2014 | * | * | * | * | * | * | * | * | 8 |
| Kabat et al., 2018 | * | * | * | * | ** | * | * | * | 9 |
| Liang et al., 2019 | * | * | * | * | * | * | * | - | 7 |
| Lin et al., 2021 | * | * | * | - | ** | * | * | * | 8 |

Note. The mean value for the 5 cohort studies included in this systematic review was 7.6 points, indicating that the included studies are of high quality.

**References**

[1] Jeong SM, Choi T, Kim D, et al. Association between high-density lipoprotein cholesterol level and risk of hematologic malignancy. Leukemia. 2021;35:1356-64. doi:10.1038/s41375-020-01081-5

[2] Tavori H, Ormseth MJ, Lilley JS, et al. Progressively decreasing plasma high-density lipoprotein cholesterol levels preceding diagnosis of smoldering myeloma. J Clin Lipidol. 2020;14:293-6. doi:10.1016/j.jacl.2020.04.001

[3] Liu X, Xu P, Wang L, et al. Cholesterol Levels Provide Prognostic Information in Patients with Multiple Myeloma. Clin Lab. 2020;66. doi:10.7754/Clin.Lab.2019.190824

[4] Pedersen KM, Colak Y, Bojesen SE, Nordestgaard BG. Low high-density lipoprotein and increased risk of several cancers: 2 population-based cohort studies including 116,728 individuals. J Hematol Oncol. 2020;13:129. doi:10.1186/s13045-020-00963-6

[5] Veskovski L, Andersson PO, Turesson I, Malmodin D, Pedersen A, Mellqvist UH. Serum metabolomic profiling correlated with ISS and clinical outcome for multiple myeloma patients treated with high-dose melphalan and autologous stem cell transplantation. Exp Hematol. 2021;97:79-88 e8. doi:10.1016/j.exphem.2021.02.007

[6] Choi T, Choi IY, Han K, et al. Lipid Level, Lipid Variability, and Risk of Multiple Myeloma: A Nationwide Population-Based Study of 3,527,776 Subjects. Cancers (Basel). 2021;13. doi:10.3390/cancers13030540

[7] Chen MZ, Zhang XY, Wang ME, Huang RF, Fan CM. [Serum Lipid Levels and Their Prognostic Significance in Patients with Multiple Myeloma]. Zhongguo Shi Yan Xue Ye Xue Za Zhi. 2022;30:1162-9. doi:10.19746/j.cnki.issn.1009-2137.2022.04.029

[8] Tsai LY, Tsai SM, Lee SC, Liu SF. Falsely low LDL-cholesterol concentrations and artifactual undetectable HDL-cholesterol measured by direct methods in a patient with monoclonal paraprotein. Clin Chim Acta. 2005;358:192-5. doi:10.1016/j.cccn.2005.02.008

[9] Aviram M, Carter A, Brook JG, Tatarsky I. Chylomicronaemia in multiple myeloma. Scand J Haematol. 1985;34:436-41. doi:10.1111/j.1600-0609.1985.tb00774.x

[10] Gozzetti A, Gennari L, Merlotti D, et al. The effects of zoledronic acid on serum lipids in multiple myeloma patients. Calcif Tissue Int. 2008;82:258-62. doi:10.1007/s00223-008-9123-8

[11] Wang H, Chen B, Shao R, et al. A new prediction model integrated serum lipid profile for patients with multiple myeloma. J Cancer. 2022;13:1796-807. doi:10.7150/jca.69321

[12] Ozturk E. The Relationship Between Hematological Malignancy and Lipid Profile. Medeni Med J. 2021;36:146-51. doi:10.5222/MMJ.2021.91145
